# Hashimoto’s encephalopathy: an endocrinological point of view

**DOI:** 10.3389/fendo.2024.1367817

**Published:** 2024-04-10

**Authors:** Laura Croce, Marzia Dal Molin, Marsida Teliti, Mario Rotondi

**Affiliations:** ^1^ Department of Internal Medicine and Therapeutics, University of Pavia, Pavia, Italy; ^2^ Unit of Endocrinology and Metabolism, Laboratory for Endocrine Disruptors, Istituti Clinici Scientifici Maugeri Istituto di Ricovero e Cura a Carattere Scientifico (IRCCS), Pavia, Italy

**Keywords:** Hashimoto’s encephalopathy, thyroid, autoimmunity, anti-thyreoperoxidase antibodies, anti-thyroglobulin antibodies

The first case of Hashimoto’s encephalopathy (HE) reported in literature, dates back to 1966, when Lord Brain described a case of a 48-year-old patient presenting with the association between neurological symptoms and autoimmune thyroiditis, suggesting a link between these clinical conditions (1).

Following this first description, HE is currently defined as a rare condition characterized by encephalopathy and central nervous system dysfunction, in the absence of detectable infection and/or structural abnormalities of the Central Nervous System (CNS), in the presence of positive tests for thyroid auto-antibodies (auto-Ab), either anti-thyreoperoxidase antibodies (TPO-Ab) or anti-thyroglobulin antibodies (Tg-Ab). This condition is also referred to as “Steroid-responsive-encephalopathy associated with autoimmune thyroiditis” (SREAT) in relation to the overall general responsiveness to steroid treatment.

The prevalence of HE is estimated to be around 2.1 per 100,000 (2), affecting females 4 to 5 times more than males, with an average age between 45 and 55 years (3). However, cases have also been reported in the paediatric population (4).

As for the clinical presentation, patients with HE can display a wide range of signs and symptoms, involving both the neurological and psychiatric spheres. Just to give a few examples, symptoms at presentation can include stroke-like episodes, cognitive dysfunction, tremor, psychosis, paranoia, hallucinations, altered consciousness, transient aphasia, seizures, myoclonus, gait disorder or ataxia and focal deficits (5).

In the search for recurring patterns in such heterogeneous presentation, a classification based on two main phenotypes has been proposed. The “vasculitic type”, in which the predominant presentation includes episodes mimicking strokes with focal neurological deficits. Confusion and cognitive deficits may or may not be present, sometimes in association with epileptic seizures; the clinical course is relapsing/remitting. On the other hand, the “diffuse progressive type”, characterized by a more insidious onset and a worsening course, accompanied by progressive cognitive deterioration, including dementia, drowsiness, and psychosis may also be observed (1). The two types may overlap, particularly in the long-term course and when left untreated. In both cases, the prognosis is generally favourable when adequately treated.

As far as the diagnosis is concerned, it is crucial to highlight that HE is mainly a diagnosis by exclusion. In 2016, Graus et al. proposed 6 criteria which must be simultaneously satisfied in order to render a diagnosis of HE (6). Although the original Graus’s criteria encompass altered thyroid function, either subclinical or overt, it should be argued that, according to currently available literature data, up to 30% of patients with HE were euthyroid at presentation (7). Accordingly, a modification of the original criteria was proposed by the same authors (8) as shown in **Table 1**.

**Table 1:** Diagnostic criteria for Hashimoto’s Encephalopathy.

PANEL A: Graus criteria
1. Encephalopathy with seizures, myoclonus, hallucinations, or stroke-like episodes
2. Subclinical or mild overt thyroid disease (usually hypothyroidism) *
3. Brain MRI normal or with non-specific abnormalities
4. Presence of serum thyroid (thyroid peroxidase, thyroglobulin) antibodies (without disease-specific cutoff value for these antibodies)
5. Absence of well characterized neuronal antibodies in serum and CSF
6. Reasonable exclusion of alternative causes
PANEL B: revised Graus criteria
7. Subacute onset of cognitive impairment, psychiatric symptoms, or seizures
8. Euthyroid status or mild clinical or subclinical hypothyroidism
9. High serum TPOAb >200 IU/mL
10. Absent neuronal antibodies in serum and CSF
11. No evidence of infectious, toxic, metabolic, vascular, or tumoral causes that could explain the symptoms

HE represents a controversial condition and its pathogenesis is still largely unknown. Knowledge about the pathology is based mainly on case reports and literature reviews (9–11). Several more or less convincing hypotheses have been proposed overtime, however a definitive elucidation is yet to be unveiled, also in view of the limited data available from both autopsy studies or animal models. The most consistent hypotheses would involve autoimmune CNS vasculitis with or without immune complex deposits (1) or a autoimmune reaction to antigens shared by the thyroid gland and the CNS (12). Cases of coexistent demyelination have also been reported (13). In addition, some authors suggested a role of a toxic effect exerted by thyrotropin-releasing hormone (TRH) in the CNS, but definite proof is lacking (14).

By definition, positive tests for circulating thyroid autoantibodies, particularly TPO-Ab, must be present. Tg-Ab may be absent, being positive in about 70% of cases (7). Despite the presence of positive tests for thyroid auto-Ab is a mandatory criteria for diagnosing HE, it seems worth highlighting that there is no correlation between thyroid autoantibody titres and the severity of HE (7). Furthermore, it should be remembered that positive TPO-Ab and Tg-Ab are very frequently detected even in the general population, with epidemiological studies suggesting that more than 10% of healthy individuals have detectable anti-thyroid antibodies (15, 16). Thus, one could argue that the crucial factor for diagnosing HE (i.e. positive thyroid auto-Ab) would be characterized by a rather low specificity. This statement would be strengthened by the findings of a recent study showing that, among 74 patients with a suspicion for autoimmune encephalitis, 8% of patients had positive TPO-Ab with a high titre, thus encountering the definition of HE. Nevertheless, the TPO-Ab positivity rate was similar between this group and a control group of patients with several well-established neurological conditions, including Multiple Sclerosis, neuromyelitis optical and psychiatric disorders (8). This notion further reduces the usefulness of circulating anti-thyroid antibodies in serum for differential diagnosis in the context of neurological diseases. A further important aspect derived from the study by Mattozzi et al, stems from the demonstration that only 31% of patients fulfilling all the criteria for HE fully responded to steroid therapy, which would contrast with the above stated alternative term for referring to HE (i.e. SREAT) (8).

Lastly, it should be acknowledged that most of the HE cases in literature were described before the widespread use of neuronal surface antibodies measurement in Autoimmune Encephalitis (17–19). These antibodies are probably pathogenetic and disease-specific, and identify patients that are usually well-responsive to steroids. It is thus probable that many early HE cases would be classified nowadays as neuronal-surface Ab positive encephalitis (20). Up to now, identified neuronal surface antibodies include, for example, anti-N-methyl-D-aspartate receptor (anti-NMDAR), anti-leucine-rich glioma-inactivated protein 1 (anti-LGI1), anti-contactin-associated protein-like 2 (anti-CASPR2), and anti-gamma-amino butyric acid B receptor (anti-GABAb) (21, 22). Nevertheless, having positive neuronal surface antibodies is not a mandatory criteria for diagnosing autoimmune encephalitis, and many patients are still classified as “serum-negative”. These patients are not well characterized and often display a worse response to commonly employed immune-modulating therapies (23). Thus, this is still a rapidly developing field, and the better characterization of CNS-specific antibodies will probably allow in the future a more satisfactory classification.

As a further aspect to be discussed, some recent studies suggest that patients with Hashimoto’s thyroiditis, even if euthyroid, can display subtle alterations in central nervous system function, including alterations in brain bioelectrical activity, as assessed by visual and brainstem auditory evoked potentials (24) or magnetic resonance spectroscopy (25), as well as mild cognitive dysfunction. These data would suggest that mild neurologic alterations could occur in patients with HT even in absence of an overt thyroid dysfunction, and could represent a very mild form of HE. However, at present, no clinical study ever evaluated whether levothyroxine therapy in euthyroid patients with HE could provide any clinical benefit.

In this context, some Authors have evaluated the possible role of thyroid autoantibodies in the Cerebro-Spinal Fluid (CSF) as a diagnostic and pathogenetic marker (26, 27). In a 2003 Italian study, TPO-Ab and Tg-Ab were evaluated in the sera of 91 patients referring to Emergency for a wide range of acute neurological states of unknown origin. In the 6 patients showing positive tests for circulating Tg -Ab and TPO-Ab, these latter were also measured in the CSF. The same tests were run in a control group of 21 patients with well-established neurological conditions. The results showed that Tg-Ab and TPO-Ab were absent in the CSF of all controls, despite the clear presence of a blood–brain barrier dysfunction in some cases, while the same antibodies could be measured in the CSF of all the 6 patients with a suspect of HE. The Authors stated that the marked increases in Tg-Ab and TPO-Ab antibody-specific indices (according to the formula AbCSF/AbSerum : IgGCSF/IgGSerum) and the normal albumin concentration in the CSF suggested the integrity of the blood–brain barrier and the possible intrathecal synthesis of autoantibodies. Nevertheless, it should be highlighted that positive anti-thyroid antibodies in the CSF were also reported in cohorts of patients with unipolar depression and schizophrenia (28, 29).

In order to establish a diagnosis of HE, the patient should undergo to a set of tests commonly conducted to exclude organic dysfunctions of the central nervous system, even if there is no consensus regarding the array of screening procedures that should be employed.

Firstly, routine blood tests are conducted to check thyroid function and the possible presence of autoantibodies. Patients are then subjected to a brain Magnetic Resonance Imaging, which, in about 50% of cases, does not show significant alterations. In the remaining cases, there may be nonspecific changes, such as generalized cerebral atrophy, diffuse increased signal on T2-weighted and FLAIR images in subcortical white matter, and dural enhancement (30).

Electroencephalography (EEG) is also not very useful in the diagnosis of HE, as it often shows nonspecific alterations. However, a common finding is represented by generalized slow-wave abnormalities of the background activity, reflecting the severity of the underlying encephalopathy (31).

Over time, the use of alternative serum markers has been hypothesized, such as autoantibodies against the amino terminal of alpha-enolase (32). However, these markers turned out to lack specificity, being associated, for example, with Creutzfeldt-Jakob disease (33).

Of particular relevance, a recent study from the Autoimmune Neurology Clinic at the Mayo-Clinic retrospectively evaluated 144 patients with an initial diagnosis of HE over a 13-year span. All patients, by definition, had positive thyroid antibodies (TPO-Ab, Tg-Ab, or both); of these, 72% were women. Demographic and clinical characteristics, laboratory results including autoimmune serological evaluation, cerebrospinal fluid analysis, neuroimaging, EEG results, cognitive test data and final clinical diagnoses were reviewed. Interestingly, throughout the study span, 105 out of 144 (73%) received an alternative diagnosis including neurodegenerative disorders, functional neurological disorders, cognitive disorders, primary psychiatric disorders, other medical conditions causing secondary encephalopathy, or non-autoimmune aetiologies of epilepsy. This study emphasizes how the lack of a specific clinical syndrome and specific diagnostic test abnormalities can contribute to raise the rate of patients receiving a diagnosis of HE. In other words, the more we search for alternative causes, the less we diagnose HE!

The first-line pharmacological treatment involves the administration of high-dose corticosteroids, although there are no specific guidelines regarding dose and duration. A commonly used regimen is the use of intravenous methylprednisolone at a dose of 500–1000 mg for a week, followed by oral prednisone at a dose of 1–2 mg/kg/day for six to eight weeks, with a gradual tapering of the steroid dose after recovery (34).

In cases of no response to steroid treatment, patients who do not tolerate corticosteroids, or those who relapse during steroid tapering, the use of other immunosuppressive drugs such as Azathioprine, Rituximab, Methotrexate, or Mycophenolate Mofetil should be considered (35). Other agents currently employed in an experimental setting are monoclonal antibodies targeting B cells, the complement cascade, the neonatal Fc receptor and IL-6 (36).

In recent years, a therapeutic approach using the administration of immunoglobulins was described, with a good outcome (37). On the other hand, plasma exchange has also been attempted, but the results have been clinically discouraging despite TPO-Ab falling to levels below the limits of laboratory detection, making the use of this technique not recommended at present (38).

As endocrinologists, we should be aware that in case of altered thyroid function, it is necessary to start specific therapies aimed at restoring euthyroidism, as well as that we do not expect relevant impact on neurological symptoms and/or on the course of encephalopathy.

In conclusion, currently available data on HE clearly indicate that: i) it is a neurological disorder; ii) the presence of positive tests for thyroid autoantibodies together with the female gender prevalence would be in line with an autoimmune etiology; iii) the lack of any relationship between HE severity and TPO-Ab/Tg-Ab titers and the persistency of neurological symptoms following thyroid Ab reduction makes it reasonable to regard thyroid auto Ab as an epiphenomenon rather than as a causative factor; iv) the fact that a certain percentage patients fail to respond to corticosteroids would support the concept that HE encompasses a wider spectrum of conditions than previously thought; v) the possibility of a serum negative autoimmune encephalitis has not been systematically ruled out.

As a last point, 58 years passed since the first description of HE. Notably, even if diagnostic procedures (i.e. neuroimaging, biochemical assay, genetic testing …) have greatly ameliorated, the fact that HE continues being diagnosed indicates that we are not at the end.

## Author contributions

LC: Writing – original draft, Methodology, Data curation. MD: Writing – original draft. MT: Writing – review & editing. MR: Writing – review & editing, Validation, Supervision, Conceptualization.
